# Circulating MicroRNA Biomarkers in Melanoma: Tools and Challenges in Personalised Medicine

**DOI:** 10.3390/biom8020021

**Published:** 2018-04-26

**Authors:** Sophie L. Mumford, Benjamin P. Towler, Amy L. Pashler, Onur Gilleard, Yella Martin, Sarah F. Newbury

**Affiliations:** 1 Medical Research Building, Brighton and Sussex Medical School, University of Sussex, Falmer, Brighton BN1 9PS, UK; s.mumford@bsms.ac.uk (S.L.M.); b.towler@bsms.ac.uk (B.P.T.); a.l.pashler@bsms.ac.uk (A.L.P.); 2 Pathology and Pharmacy Building at The Royal London Hospital, 80 Newark Street, London E1 2ES, UK; onurgilleard@aol.com; 3 Huxley Building, School of Pharmacy and Biomolecular Sciences, University of Brighton, Lewes Road, Brighton BN2 4GJ, UK; y.martin@brighton.ac.uk

**Keywords:** melanoma, microRNAs, circulating biomarkers, diagnostic biomarkers, prognostic biomarkers, exosomes

## Abstract

Effective management of melanoma depends heavily on early diagnosis. When detected in early non-metastatic stages, melanoma is almost 100% curable by surgical resection, however when detected in late metastatic stages III and IV, 5-year survival rates drop to ~50% and 10–25%, respectively, due to limited efficacy of current treatment options. This presents a pressing need to identify biomarkers that can detect patients at high risk of recurrence and progression to metastatic disease, which will allow for early intervention and survival benefit. Accumulating evidence over the past few decades has highlighted the potential use of circulating molecular biomarkers for melanoma diagnosis and prognosis, including lactate dehydrogenase (LDH), S100 calcium-binding protein B (S100B) and circulating tumor DNA (ctDNA) fragments. Since 2010, circulating microRNAs (miRNAs) have been increasingly recognised as more robust non-invasive biomarkers for melanoma due to their structural stability under the harsh conditions of the blood and different conditions of sample processing and isolation. Several pre-analytical and analytical variables challenge the accurate quantification of relative miRNA levels between serum samples or plasma samples, leading to conflicting findings between studies on circulating miRNA biomarkers for melanoma. In this review, we provide a critical summary of the circulating miRNA biomarkers for melanoma published to date.

## 1. Introduction

Melanoma is an aggressive cancer derived from melanocytes found predominantly in the skin, but also in the eyes, ears, gastrointestinal tract, and oral and genital mucosal membranes. The global incidence of melanoma has increased dramatically over the past few decades. In the UK alone, melanoma incidence rates have increased by a staggering 128% since the early 1990s, rendering melanoma the UK’s fifth most common cancer with around 15,906 new cases each year. While melanoma accounts for only 4% of new skin cancer cases in the UK each year, it is the cause of more than 95% of skin cancer-related deaths, and is considered the most common fatal malignancy of young adults [1].

When detected in the early non-metastatic stages I and II, melanoma has a 5-year survival rate of ~100% and 80–90%, respectively, as thin localized tumors are highly curable by surgical resection. While surgical resection of a non-metastatic melanoma can result in disease-free intervals, recurrence is common often resulting in progression to late metastatic stages III and IV, with a 5-year survival rate of ~50% and 10–25%, respectively [2]. This presents a pressing need to identify biomarkers that allow the detection of melanoma in its earliest stages, and identify patients at the highest risk of metastatic recurrence. Early detection will maximize the chances of patient survival, as there are now several treatment options available that are effective against certain subtypes of stages I, II, and III melanoma [3]. These include the use of new targeted treatments, such as BRAF inhibitors [4], and immunological approaches using the anti-CTLA4 antibody Ipilimumab [5], which can improve life expectancy.

Diagnosis of melanoma currently requires the removal and analysis of the primary melanoma, and if high risk markers are detected, a sentinel lymph node biopsy to determine the presence and stage of metastatic disease [6]. This is invasive, expensive, and time consuming, therefore, given the clinical importance of melanoma, several research laboratories have made great efforts to identify non-invasive blood-based biomarkers which will allow economical, rapid, and repeat sampling and therefore earlier intervention and dynamic treatment management. Circulating biomarkers such as lactate dehydrogenase (LDH) and S100 calcium-binding protein B (S100B) have some prognostic value but do not translate into an adequate therapeutic intervention and survival benefit [7]. For example, high levels of LDH in the serum of melanoma patients is indicative of current (as opposed to predicted) progression to late stage metastatic disease [8,9] and there is limited efficacy of current treatment options against both stages III and IV melanomas [3]. Increasing concentrations of serum S100B is indicative of disease progression, with a moderate increase in concentration between stages I–III melanoma patients and a dramatic increase in concentration between stages III–IV melanoma patients [10,11,12], by which time survival rates have already dropped to 10–25% [2]. Therefore, circulating biomarkers which can be used at earlier stages of melanoma could be of immense benefit to patient survival.

The presence of circulating tumor DNA (ctDNA) fragments have been correlated with overall tumor burden in melanoma [13,14] and have therefore emerged as promising non-invasive biomarkers. Detection of ctDNA fragments relies on the identification of specific, known genetic alterations derived from mutations, chromosomal rearrangements, and copy number variations and amplifications, therefore tumor heterogeneity continues to challenge this field. The precise mechanism of ctDNA release is unclear; however, it is thought that ctDNA is released primarily through intermittent apoptosis or necrosis [15,16,17,18] of cancer cells resulting in low levels of ctDNA quantity in circulation. Furthermore, ctDNA is relatively unstable in the blood with a half-life of less than 2 h [19]. Therefore, delays in processing the blood and the collection conditions mean that it is difficult to accurately quantify comparative expression levels between patient groups. Since the amount of ctDNA released is proportional to the size of the patient’s tumor, detection of ctDNA at early stages of cancer, when patients would most benefit from treatment, is problematic. 

Circulating microRNAs (miRNAs) are emerging as potential non-invasive biomarkers for melanoma. MicroRNAs are also released following tumor cell apoptosis or necrosis, but this is not the primary route of release into circulation. In the circulating blood, miRNAs are usually encapsulated within lipid particles, termed exosomes, and/or are bound by protective proteins, such as AGO2 and Nucleophosmin. As such, circulating miRNAs are very stable; a feature which is highly advantageous for a useful biomarker. Although miRNAs are present at extremely low concentrations in the circulating blood, they can be detected by standard techniques including real-time quantitative reverse transcription polymerase chain reaction (qRT-PCR). The altered expression of specific miRNAs observed in circulation between cancer patients and healthy controls is likely to be the result of the altered expression and active release from tumor cells and other cells (such as T-cells) within the microenvironment as opposed to intermittent release from tumor cells due to cell death. This cross-talk of miRNAs between different cells and cell types via the blood circulation is likely to be a form of cell-to-cell communication, although the mechanisms by which specific miRNAs are secreted and taken up remain unclear.

To date, several efforts have been made towards the identification of circulating miRNA biomarkers for diagnostic and prognostic utility in melanoma. However, several pre-analytical and analytical variables challenge the accurate quantification of relative miRNA levels between serum samples or plasma samples. The most prominent of these variables include serum/plasma preparation and storage, RNA extraction methods, global quantity and quality assessment, miRNA profiling platforms and normalization methods, which result in very limited consistency between studies. This review provides a critical summary of the evidence that gives credence to the utility of specific circulating miRNAs as biomarkers for detection and diagnosis of melanoma and for monitoring of melanoma disease status. 

## 2. MicroRNA Biogenesis and Function

**Figure 1:**
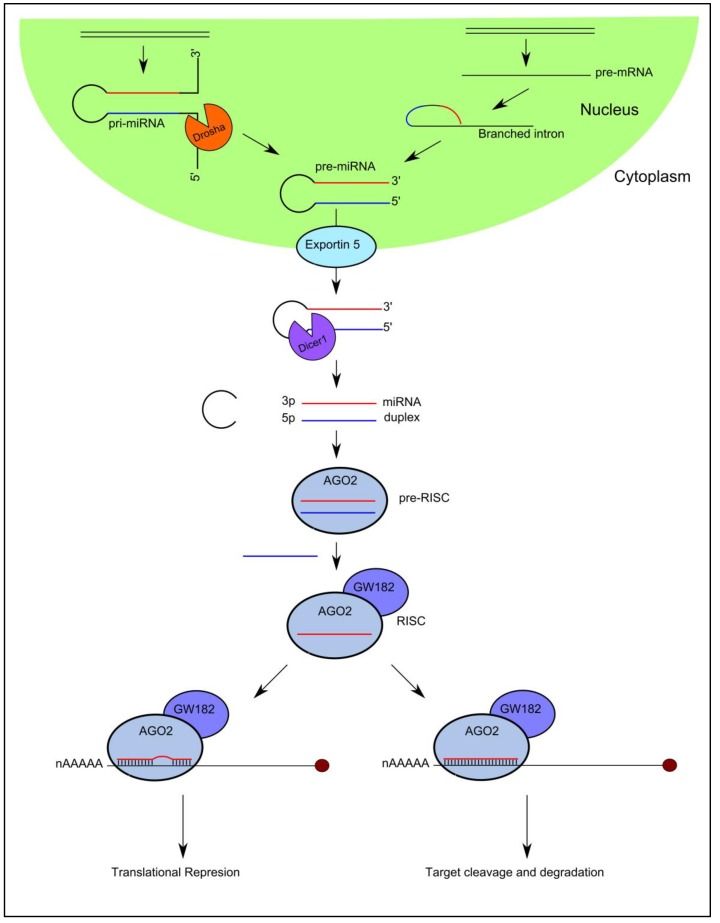
MicroRNA (miRNA) biogenesis and function. Precursor molecules termed primary miRNAs (pri-miRNAs) are transcribed from their cognate genes in the nucleus. Pri-miRNAs are cleaved by Drosha and DGCR8, to generate a 60–70 nucleotide precursor miRNAs (pre-miRNAs). Pre-miRNAs are transported to the cytoplasm by Exportin-5 in a Ran-GTP dependent manner, where it they are cleaved by Dicer generating a small miRNA duplex. TAR-RNA-binding protein TRBP interacts with Dicer and the bound double strand RNA (dsRNA) duplex, and recruits AGO2 to nucleate RNA-induced silencing complex (RISC). The guide strand is incorporated into RISC, while the complementary strand is excluded. The mature miRNA then guides RISC-induced messenger RNA (mRNA) down-regulation through translational repression or mRNA cleavage depending on the level of sequence complementarity between the miRNA and target mRNA.

High-throughput sequencing technologies have been developed over the past decade that have enabled the discovery of a diverse catalogue of small non-coding RNA molecules that function in the post-transcriptional control of gene expression by associating with Argonaute (AGO) proteins. MicroRNAs are the best characterized Ago-associated post-transcriptional regulators that follow a specific biogenesis pathway characterized by Drosha/DGCR8 and Dicer processing (Figure 1). 

In brief, capped and polyadenylated precursor molecules termed primary-miRNAs (pri-miRNAs), that can measure up to several thousand nucleotides in length and contain stem-loops in their structure, are transcribed in the nucleus from their cognate miRNA genes. They are processed by a number of nucleases including Drosha [20] and Dicer [21,22] to yield a small double stranded RNA duplex measuring 18–22 nucleotides in length [23,24,25,26,27]. One strand (guide strand) of the miRNA duplex is selected as the mature miRNA and preferentially incorporated into AGO2 in the formation of the mature RNA-induced silencing complex (RISC), while the complementary strand is excluded from the complex and subsequently degraded [28,29]. It is important to note that exotic miRNA species exist that bypass particular steps of this canonical pathway (Figure 1). 

The mature miRNA guides RISC to specific target messenger RNAs (mRNAs) through Watson-Crick base-pairing, initiating downregulation of gene expression by one of two posttranscriptional mechanisms: (i) translational repression or (ii) mRNA cleavage [23]. The degree of sequence complementarity between the miRNA and the target mRNA is thought to determine the mechanism of downregulation: the miRNA will specify mRNA cleavage only if there is sufficient complementarity between the two sequences; if there is not sufficient complementarity between the sequences, miRNA binding will result in translational repression [30]. 

There are known to be 2661 miRNAs encoded in the human genome (miRBase 21.0 [31]). Due to the imperfect sequence matching between the miRNA and target, a single miRNA can downregulate a large number direct target genes and a single mRNA can be regulated by several miRNAs [32]. In fact, it is thought that over 30% of protein coding genes in the human genome are regulated by miRNAs. It is known that miRNAs play a critical role in the regulation of genes involved in multiple cellular processes including proliferation, self-renewal, differentiation, migration, and apoptosis. It is therefore not surprising that dysregulation of the miRNA expression profile contributes to several pathologies, including inflammation, cardiovascular diseases, neurological disorders, and many types of cancer. 

## 3. MicroRNAs: Roles in Melanoma Development and Progression

MicroRNAs play a critical role in the regulation of numerous cancer-relevant processes including proliferation, migration and apoptosis, by regulating oncogenes (tumor suppressor miRNAs) or tumor suppressor genes (oncomiRs). Over the past decade, it has become increasingly clear that miRNA expression is dysregulated in human malignancies; a result of chromosomal abnormalities (i.e., insertions, deletions, and amplifications), transcriptional control changes, epigenetic changes, and defects in the miRNA biogenesis machinery [33], and that miRNA dysregulation directly contributes to the acquisition of the hallmarks of cancer, as defined by Hanahan and Weinberg [34].

A large number of miRNAs have been implicated in the development and progression of melanoma. It has been demonstrated that miR-221 and miR-222 directly target the cell-cycle regulator p27 in the Me1402/R melanoma cell line [35], and down-regulation of p27 by miR-221 and miR-222 has been shown to promote cell proliferation in the context of prostate carcinoma cell lines and glioblastoma cell lines [36,37]. It has been demonstrated that exosomes released by miR-222-overexpressing melanoma cells can confer miR-222 mediated malignancy when taken up by recipient primary melanoma cells [38]. miR-137 is a well-established tumor suppressor miRNA often downregulated in melanoma, as well as many other cancer types. MiR-137 is able to inhibit invasion and migration of melanoma cell lines by directly targeting oncogenes including the transcription factor TBX3, EZH2, c-Met, and Y box–binding protein 1 (YB1) [39,40]. It is therefore not surprising that downregulation of tumor suppressor miR-137 expression is associated with poor prognosis in melanoma patients [41]. Tumor suppressor miR-493 is frequently down-regulated in melanoma. miR-493 inhibits proliferation and cell cycle progression by directly targeting IRS4 RNA and its downregulation was shown to promote proliferation of the melanoma cell line A375 [42]. Further, it has been demonstrated that miR-7-5p, another tumor suppressor miRNA that is frequently downregulated in melanoma, inhibits melanoma cell proliferation, and metastasis by directly suppressing RelA/NF-κB [43]. miR-21 *is* an example of an oncomiR that is overexpressed in many types of cancer, including melanoma. miR-21 has been demonstrated to directly target FBXO11 RNA [44] and promote the proliferation, migration, and inhibit the apoptosis of, human melanoma A375 cells by inhibiting SPRY1, PDCD4, and PTEN, and promoting ERK/NF-κB signalling [45]. These are examples of just some of the miRNAs implicated in melanoma development and progression. A number of reviews exist, that highlight the role of miRNAs as potential diagnostic and prognostic biomarkers and key molecular regulators in the development of melanoma [46,47]. 

A major challenge in studying the role and targets of specific miRNAs in melanoma is the considerable level of redundancy due to imperfect sequence matching. While the majority of published studies focus on individual targets of a given miRNA molecule, most miRNAs are likely to exert their full functional effects on melanoma development and progression via a large cohort of target genes, and a single gene is likely to be regulated by a large cohort of miRNAs. Hence, one should be careful in drawing firm conclusions about the particular function or phenotype of any given miRNA molecule in melanoma. 

## 4. Circulating MicroRNAs: Release Mechanisms and Function

Circulating miRNAs have been detected in peripheral blood circulation and other body fluids. The expression profile of miRNAs from tumors can effectively distinguish tumors from normal tissues. Similarly, the expression profile of circulating miRNAs from biofluids in relation to different cancer types and cancer stages show specific patterns, indicating that they are selectively released from cancer cells and other cells within the tumor microenvironment, as opposed to being released primarily from necrotic or injured cells. Extracellular miRNAs are released into human bodily fluids in a remarkably stable form, sometimes with a half-life of up to 24 h [48], while ctDNA has a half-life of less than 2 h.

Currently, there are several models that can explain this stability. It is widely accepted that miRNAs are released from cells in extracellular vesicles including exosomes, microvesicles, and apoptotic bodies, which prevent degradation by serum and plasma RNases. A large portion of circulating miRNAs are not derived from extracellular vesicles; instead, they are associated with and stabilized by protective proteins, such as AGO2 and Nucleophosmin, or high density lipoproteins [49]. Exosomes are thought to have critical roles in cell–cell communication [50,51]. Melanoma cells secrete different types of extracellular vesicles including exosomes, microvesicles and apoptotic bodies [52]. It is widely accepted that exosomes, the best characterized of the extracellular vesicles, transport information in the form of regulatory RNA molecules that can modulate the activity of recipient cells. These exosomes may serve to modulate immune cell behavior, dampening the immune response and promoting melanoma progression [50,53,54,55,56,57]. Similarly, exosomes secreted by immune cells may serve to modulate melanoma cell behavior and exert therapeutic effects [58]. Thus, circulating miRNAs may reflect the homeostatic response of the organism, as well as being signs of disease progression. Owing to their stability and resistance to endogenous RNase activity, these miRNAs have been proposed as diagnostic and prognostic biomarkers for melanoma.

RNA profiling of exosomes extracted from the blood of melanoma patients and healthy controls can be used to develop diagnostic and prognostic exosome based biomarkers. Indeed, studies have demonstrated that serum exosomes can be used as predictors of response to treatment of melanoma [59]. Further, potential miRNA melanoma biomarkers have been detected from serum exosomes which will be discussed in the next section. 

## 5. Circulating MicroRNA Biomarkers in Melanoma 

Recent studies have reported significant alterations in the miRNA expression profile in the serum and plasma of melanoma patients compared to healthy controls, proposing circulating miRNAs as promising diagnostic melanoma biomarkers. In addition to its diagnostic utility, more recent evidence suggests that miRNA profiling from serum and plasma may be a useful tool for prognosis, and prediction of metastatic outcome and therapeutic response. Prognostic biomarkers should be able to differentiate between stage I/II and stage III/IV melanoma patients indicating which patients are likely to progress to metastatic disease, correlate with poor prognosis and/or stratify patients into groups at high and low risk of recurrence following surgical resection of a non-metastatic melanoma. Lack of accurate prognostic biomarkers could lead to unnecessary lymph node biopsies and lymph node resections which are invasive to the patient.

Since 2010, several efforts have been made to identify diagnostic and prognostic circulating miRNA biomarkers for melanoma, however due to the use of different profiling platforms and inputs, and variable techniques for serum and plasma preparation, RNA extraction, quality control, normalization, and statistical evaluation, reported results between studies show very limited consistency. Table 1 and Table 2 summarize circulating miRNAs found to be dysregulated in melanoma patients to date, as well as the technical variables that may lead to the lack of consistency between studies. 

### 5.1. Circulating MicroRNAs as Diagnostic Biomarkers in Melanoma

Several efforts have been made to identify potential diagnostic biomarkers that can distinguish between melanoma patients and healthy control individuals (Table 1). Leidinger et al. [60] were one of the first research groups to use high throughput screening techniques to identify diagnostic circulating miRNA biomarkers. This work involved screening ~866 human miRNAs in blood cells using a microarray-based approach, and subsequent validation of differentially expressed miRNAs using qRT-PCR. A 16-miRNA diagnostic signature including miR-186, let-7d, miR-18a, miR-145, miR-99a, miR-664, miR-501-5p, miR-378, miR-29c, miR-1280, miR-365, miR-1249, miR-328, miR-422a, miR-30d, and miR-17, that could distinguish between melanoma patients and healthy control individuals with high sensitivity and specify was identified. It is important to note that the majority of melanoma samples used for this study derived from patients with metastatic (stages III and IV) melanoma. It would be interesting to investigate this 16-miRNA signature as a diagnostic biomarker for non-metastatic (stages I and II) melanoma, against which the efficacy of current treatment options and therefore chances of survival are much higher.

More recently, Van Laar and colleagues [61] performed microarray profiling on plasma samples from 32 patients with stages I–IV melanoma and 16 healthy control individuals. They identified MEL38, a 38-miRNA signature (Table S3) that is able to distinguish between melanoma patients (stages I–IV) and healthy controls with a high degree of sensitivity and specificity. Instead of using an independent cohort of patients to validate the significance of the circulating miRNAs identified from the discovery series, a classification algorithm was trained on their discovery data and independently validated on miRNA expression data sets representing 473 unique melanoma patients, normal control individuals or cell-line models, downloaded from gene expression data repositories, including National Center for Biotechnology Information (NCBI) Gene Expression Omnibus (GEO) and European Bioinformatics Institute (EBI) ArrayExpress. A major disadvantage to this approach is the likely presence of technical differences in experimental design between the studies from which these data sets were generated. As discussed in Section 6, small changes to experimental design from sample collection and processing to data interpretation and analysis can have a significant influence on the levels of miRNA detected from plasma and serum. None of the miRNAs in MEL38 were identified as differentially expressed in the blood cell screening by Leidinger et al. [60], which is likely due to the starting material or improvements in detection technology. Nearly half of miRNAs in MEL38 displayed between a 2- and 2.5-fold change which, because the amount of RNA being input into each assay is unknown (discussed in Section 6), could indeed result from normal human variation as opposed to disease status. This highlights the benefit of using a collection of miRNAs as a signature as if used individually, many of the miRNAs in MEL38 cannot be considered as independent biomarkers, however when used in combination appear to provide a sensitive and specific tool.

miR-424-5p and miR-301a-3p from the MEL38 series displayed a 6.28- fold and 4.86- fold) increase, respectively, in melanoma patients compared to healthy controls, and can therefore be considered as potential independent diagnostic biomarkers or together form a signature with high diagnostic power. In contrast with these findings, Margue et al. [62] demonstrated that miR-301a-3p is down-regulated in stage III/IV melanoma patients relative to healthy controls, however this study used serum as opposed to plasma, and these two biofluids have been shown to differ in their miRNA expression profile, perhaps due to different sample processing and RNA extraction methods inadvertently enriching for particular miRNAs (discussed in Section 6). In fact, several miRNAs in the MEL38 discovery series were found differentially expressed in previously published melanoma circulating miRNA signatures, including miR-424-5p [63], miR-205-5p, miR-301a-3p, and miR-27a-3p [62], however only miR-424-5p showed the same direction of change between studies indicating one of many difficulties in the field.

Several efforts have been made towards the identification of circulating miRNA biomarkers that can distinguish between stage III/IV melanoma patients and healthy controls. Margue et al. [62] used “whole” miRNome (1066 miRNAs) and custom quantitative reverse transcriptase polymerase chain reaction (qRT-PCR) arrays to identify and validate potential serum diagnostic miRNA biomarkers that can distinguish between melanoma patients (stages I–IV) and healthy control individuals. MiR-204-5p, miR-182-5p, miR-301a-3p, miR-200c-3p, miR-28-5p, miR-27a-3p, miR-197-3p, and miR-374a-5p were identified as potential independent diagnostic biomarkers down-regulated in the serum of melanoma patients relative to healthy controls, while miR-193b-3p, miR-720, miR-205-5p, miR-126-5p, miR-211-5p, miR-720, miR-206, miR-550a-3p, miR-627-5p, and miR-629-5p were shown to be up-regulated. MiR-301-3p, miR-200c-3p, miR-126-5p, miR-374a-5p, and miR-211-5p could distinguish between non-metastatic melanoma (stage I/II) patients and healthy controls, whilst the remaining miRNAs could only distinguish between metastatic (stage III/IV) melanoma patients and healthy controls. In agreement with these findings, miR-200c-3p was also demonstrated by Fogli and colleagues [64] to be down-regulated in the plasma of stage III/IV melanoma patients compared to stage I/II melanoma patients and healthy controls, suggesting its potential to be used as both a diagnostic and prognostic biomarker. It has been demonstrated that miR-200c-3p levels are significantly reduced in metastatic melanoma tissue, suggesting that lower serum/plasma levels of this miRNA may be caused by decreased expression and release from the tumor site [65]. Greenberg et al. [66] reported miR-29c-5p and miR-324-3p to be lower in the serum of metastatic melanoma patients (stage IV) compared to healthy control individuals. In addition to being indicative of metastatic melanoma; these miRNAs could distinguish between melanoma and both colon and renal cancer. It is important to note that the discovery cohort consisted of only males, and expression analysis studies have revealed potential sex-specific dysregulated miRNA expression [67]. Diagnostic biomarkers that can distinguish between stage III/IV melanoma patients and healthy control individuals, while providing a less invasive means of diagnosing metastatic melanoma, are unlikely to translate into adequate therapeutic intervention and survival benefit due to the limited efficacy of current treatment options against certain subtypes of stage III/IV melanoma (5-year survival rate of 10–50%), therefore studies focusing on diagnostic biomarkers which aim to identify patients with stage I/II melanoma (5-year survival rate of 80–100%), could improve early intervention.

Indeed, a number of studies have identified potential circulating miRNA biomarkers that can distinguish between stage I/II melanoma patients and healthy controls. It has been demonstrated that miR-15b-5p, miR-149-3p, and miR-150-5p are upregulated, and miR-193a-3p and miR-524-5p are downregulated in the plasma of stage I/II, stage III, and stage IV melanoma patients compared to healthy control individuals [64]. These miRNAs have the potential to be used as independent diagnostic biomarkers in the earlier stages of melanoma, however, diagnostic sensitivity and specificity was greatly improved when a signature of miR-149-3p, miR-150-5p, and miR-193a-3p was considered. Both the Fleming et al. [68] and Friedman et al. [63] studies (discussed in Section 5.2) support these findings by implicating increased serum miR-150 in a prognostic model of melanoma recurrence. The Fleming et al. study [68] also agreed with the increasing levels of serum miR-15b, however the Friedman et al. [63] study disagreed, implicating decreased serum miR-15b in a prognostic model of melanoma recurrence. MELmiR-7 is a serum based 7-miRNA (miR-16, miR-211-5p, miR-4487, miR-4706, miR-4731, miR-509-3p, and miR-509-5p) signature that was shown to be able to discriminate between stage I/II, stage III, and stage IV melanoma patients and non-melanoma controls [69]. MiR-4487, miR-4706, miR-4731, miR-509-3p, and miR-509-5p showed lower expression in melanoma patients whilst miR-16 and miR-211-5p had higher expression in melanoma patients. The fold changes observed in this study are more suitable for clear and consistent biomarkers (i.e., miR-509-3p displayed a ~6 ΔCq/~64-fold decrease in stage IV melanoma patients relative to healthy controls).

Currently only one study has specifically examined the exosome associated miRNA pool between melanoma patients and controls. Alegre and co-workers [70] demonstrated that miR-125b levels were significantly lower in the serum exosomes of patients with advanced melanoma compared to serum exosomes of disease-free patients and healthy controls, however no significant difference was observed in miRNAs from whole serum of melanoma patients relative to healthy controls. Conversely, Achberger et al. [71] demonstrated that plasma levels of miR-125b are upregulated in uveal melanoma patients relative to healthy controls and that miR-125b is also present at higher levels at metastasis compared to primary diagnosis. Indeed, plasma and serum have been shown to have different miRNA profiles, perhaps due to particular miRNAs levels being sensitive to extraction conditions during serum/plasma preparation or exosome enrichment. Achberger et al. [71] also demonstrated that miR-20a, miR-146a, miR-155, miR-181a, and miR-223 are upregulated in the plasma of uveal melanoma patients relative to healthy control individuals and all, except for miR-181a, are present at higher levels at metastasis compared to primary diagnosis. Whilst promising, only six uveal melanoma patients were used for this study therefore there is clear need to perform a validation study with increased sample size before ratifying the use of these circulating miRNAs as diagnostic biomarkers for melanoma.

### 5.2. Circulating MicroRNAs as Prognostic Biomarkers in Melanoma

While many studies have focused on identifying circulating miRNA biomarkers that can distinguish between melanoma patients and healthy control individuals, relatively few studies have used staged samples to identify prognostic biomarkers that can distinguish between stage I/II melanoma patients and stage III/IV melanoma patients. There is limited efficacy of current treatment options against certain subtypes of stage III and IV melanoma, resulting in 5-year survival rates of ~50% and 10–25%, respectively, therefore prognostic biomarkers that can identify patients at high risk of recurrence and progression to metastatic disease will allow for early intervention before melanoma cells have metastasized, as there is high efficacy of current treatment options against non-metastatic melanoma subtypes. This section summarizes the potential prognostic circulating miRNA biomarkers identified to date (Table 2).

Margue et al. [62] (Section 5.1) showed that an increase in levels of serum miR-193b-3p and miR-720 could not only distinguish between melanoma patients and healthy control individuals, but also between non-metastatic and metastatic melanoma patient groups, highlighting the prognostic potential of these two miRNAs. In addition to diagnostic signature, MEL38, Van Laar et al. [61] identified MEL18, an 18-miRNA serum signature that is able to distinguish between non-metastatic (stage I/II) and metastatic (stage III/IV) melanoma patients (see Table S3). Guo et al. [72] demonstrated that miR-16 is downregulated by in the serum of stage I/II melanoma patients relative to healthy controls, and is also downregulated in stage III/IV melanoma patients relative to stage I/II melanoma patients demonstrating the potential for this miRNA to be used as both a diagnostic and prognostic biomarker. MiR-16 levels were shown to negatively correlate with ulceration and tumor thickness and positively correlate with patient survival, implying that serum miR-16 levels could reflect progression status. It is important to note that both the Van Laar et al. [61] and the Guo et al. [72] studies failed to use a suitable endogenous normalization method. Further, circulating miR-16 levels are influenced by stress, therefore it would not appear to be suitable to use as a biomarker [73]. In contrast with the findings by Guo et al. [72], the Stark et al. study [69] (Section 5.1) demonstrated an increase (rather than a decrease) in miR-16 expression in the serum of stage I, II, III, and IV melanoma patients relative to healthy controls. There are a number of variations in the methodology used between these studies that may account for these conflicting results (Tables S1 and S2). For example, different miRNA isolation kits were used and it has been demonstrated that the miRNA extraction kit can influence the yield and distribution of miRNA detected from serum and plasma samples (Section 6). Furthermore, the Stark et al. [69] study used TaqMan microRNA qRT-PCR assays to measure relative expression while the Guo et al. study [72] used SYBR-Green-based qRT-PCR. Several studies have reported lower sensitivity, specificity and reproducibility of SYBR-Green based qRT-PCR systems relative to TaqMan qRT-PCR systems (Section 6) and that the TaqMan microRNA qRT-PCR system can minimize extraction kit-dependent variation within the same set of samples [74]. Different qRT-PCR chemistries can lead to conflicting findings between studies, therefore consideration should be given to the qRT-PCR chemistry used before drawing firm conclusions about the roles of specific miRNA as circulating biomarkers in melanoma.

Several efforts have been made towards the identification of circulating miRNA biomarkers that can stratify patients into groups at high and low risk of recurrence following surgical resection. Fleming et al. [68] demonstrated that serum levels of miR-150, miR-15b, miR-425, and miR-30d increase with increasing melanoma stages, and that this miRNA signature in combination with stage can effectively distinguish recurrent cases from non-recurrent cases and stratify patients into groups at high and low risk of recurrence with high sensitivity. Further, miR-15b levels were demonstrated to increase over time from pre- to post-recurrence. Friedman et al. [63] identified a five miRNA signature that includes miR-150-5p and miR-15b-5p in combination with miR-199a-5p, miR-33a-5p, and miR-425-5p that is also able stratify patients into groups at high and low risk of recurrence with high sensitivity and specificity. In agreement with the Fleming et al. [68] study, miR-150-5p was upregulated and in the serum of melanoma patients at high risk of recurrence, while in contrast, miR-15b-5p was down-regulated in the serum of patients at high risk of recurrence. Results obtained for miR-15b-5p by Fleming et al. [68] are perhaps the most reliable because they used two endogenous normalizers selected using the NormFinder algorithm, while Friedman et al. [63] used median normalization. The extraction kit and qRT-PCR chemistry were the same for both studies.

Tian et al. [75] demonstrated that levels of miR-206 were significantly lower in the serum of melanoma patients relative to healthy controls and that decreased serum miR-206 levels are associated with aggressive disease progression and poor prognosis, suggesting its potential to be used as a prognostic biomarker. miR-221 and miR-21 have been shown to be present at significantly high levels in the serum and plasma, respectively, of melanoma patients relative to healthy controls [76,77,78]. High expression of serum miR-21 matches high miR-21 expression in metastatic melanoma tissues and an increase in miR-21 from controls through to advanced melanoma stages suggests this miRNA has possible prognostic value. Further, when preoperative and postoperative cases were compared, there was a drop in plasma miR-21 levels in post-operative samples [76]. While no significant difference in miR-221 expression was observed among patients with stages I, II, III, and IV melanoma, miR-221 levels were shown to correlate with tumor thickness [77,78] which is a crucial clinicopathologic factor determining melanoma progression, implying that serum miR-221 levels could reflect progression status of melanoma patients. By assigning patients into low expression (less than the median expression level of 2.95) and high expression (≥2.95) groups, Li et al. [78] demonstrated that miR-221 expression correlates with a number of clinicopathological characteristics including tumor thickness, poor differentiation, higher T classification, higher N classification, metastasis, and advanced clinical stage. Furthermore, patients with high serum miR-221 levels had a significantly lower 5-year rate and recurrence free survival (RFS) rate than those with low serum miR-221 level. In support of these findings, Margue et al. [62] demonstrated that miR-221-5p was one of four miRNA upregulated in the serum of stage III/IV melanoma patients relative to healthy controls. miR-221 has long been recognized as marker miRNA for the melanocytic lineage [79,80] suggesting that this miRNA may be specifically secreted from melanoma cells. Furthermore, miR-221 is an established oncomiR that has been demonstrated to target p27 RNA in melanoma cell lines. miR-221-mediated down-regulation of p27 has been demonstrated to drive cell proliferation and contribute to cancer development and progression in a number of contexts (Section 3). Taken together these findings would suggest that miR-221 is a potential diagnostic and prognostic circulating miRNA biomarker for melanoma. Whilst promising, all three studies investigating circulating miR-221 as a potential prognostic biomarker for melanoma failed to specify a suitable endogenous normalization method (Table 2). Further studies, using appropriate endogenous normalizers or other suitable methods (such as the global mean normalization) [81], should be carried out before ratifying the use of increased circulating miR-221 levels as a biomarker for melanoma diagnosis and prognosis.

It has been demonstrated that the expression of mir-210, a miRNA known to play important roles in the tumor hypoxic state, to be significantly higher in the plasma of stage III/IV melanoma patient’s relative to healthy controls [82]. In the training cohort, this study demonstrated that miR-210 levels were higher in the serum of stage IV melanoma patients relative to stage III melanoma patients, however they were unable to verify this difference in the validation study. Levels of miR-210 in plasma significantly increased prior to recurrence, and correlated with poor prognosis, suggesting that miR-210 expression could provide early identification of melanoma recurrence. This study confirmed miR-210 expression level was significantly higher in both lymph node metastasis and distant organ metastasis compared to primary tumors demonstrating the development of the hypoxic miR-210 relevance to melanoma metastasis occurrence. Unlike other studies on circulating miRNA biomarkers in melanoma and other cancers, reverse transcription was carried out directly against plasma using a miRNA-specific reverse-transcriptase (RT) primer and Moloney murine leukemia virus reverse transcriptase. The concentration of RNA in serum and plasma is extremely low compared to that of RNA purified and eluted in a relatively small volume of RNase free water, which would lead to high quantitation cycle (Cq) values and broad variation within patient groups due to more stochastic qPCR amplification. This can result in the detection of false positive differences in miRNA expression between patient groups. 

## 6. Limitations of Using Circulating MicroRNA as Cancer Biomarkers

The establishment of a panel of circulating miRNAs that can be used for melanoma diagnosis, prognosis, and predication of response to treatment is challenging at almost every step from sample collection and processing to data interpretation and analysis. Variability at each step often results in very limited consistency between studies. Several critical voices have summarized the technical and biological challenges of circulating miRNA profiling studies [83,84,85,86,87,88,89,90,91,92,93,94], highlighting the lack of consistency among published circulating miRNA signatures. 

Table 1 and Table 2, together with Tables S1 and S2, compare the pre-analytical and analytical variables between studies on circulating miRNA biomarkers in melanoma. Consideration is given in most studies, to the demographic and clinical features that may influence circulating miRNA levels in patients and healthy controls, and these are generally well matched in most categories. The samples size of patient and healthy groups vary greatly between studies. For example, the Achberger et al. [71] study used only 6 melanoma study subjects and 26 healthy donors, whilst Fleming et al. [68] used 283 melanoma study subjects. A decrease in sample size increases the likelihood of detecting false positives in miRNA expression between patient groups, and therefore decreases the power of the study to draw firm conclusions about the role of miRNA of interest as circulating melanoma biomarkers.

A major limitation of using circulating miRNAs as biomarkers for melanoma is their low abundance. There are a number of methods for measuring RNA yield and purity following extraction; however, the concentration of RNA extracted from biofluids are often below the accurate detection limit. This means that the amount of RNA being input into each assay is unknown, rather a fixed volume is typically used. Circulating miRNA profiling from the serum and plasma of melanoma patients therefore relies on the assumption that total miRNA levels are consistent between samples. The low abundance of miRNAs can also hamper their detection using many standard profiling techniques. Because the measurement of miRNAs is inaccurate at very low levels due to stochastic effect, it can be advisable to use a Cq cut-off e.g., Cq > 32 (we routinely use Cq > 35).

Another important limitation is sample collection, processing and storage. Plasma and serum are the most frequently used biofluids for circulating melanoma miRNA biomarker studies. It has been demonstrated that the miRNA expression profile detected from plasma and serum can differ significantly, despite these two biofluids being differentiated only by the absence and presence respectively of coagulation factors [83]. The time between blood collection from the patient, storage at room temperature in the clinic or during transport and isolation of serum or plasma, as often occurs in clinical routine, can lead to the release of miRNAs from blood cells and therefore alter the yield and spectrum of miRNA detected from plasma and serum preparations [95]. Despite this, no studies detailed the length of time between blood samples collection and isolation of serum and plasma. Further, different centrifugation protocols can lead to different levels of cellular and platelet contamination [84]. The centrifugation protocol varies greatly between studies on circulating miRNA biomarkers in melanoma (Tables S1 and S2) and the majority of studies did not to subject their samples to an additional filtration or centrifugation step to remove remaining cellular contaminants and debris. Most studies also omitted to check and eliminate samples with high levels of hemolysis, including two studies investigating miR-16, a miRNA highly abundant in blood cells [96], as a potential biomarker for melanoma. This highlights the importance of rigorous quality control of plasma and serum samples used for measurement of circulating microRNAs. The different miRNA expression patterns detected from serum and plasma [83] may reflect these variables in sample collection, processing, and storage. Some studies have reported higher miRNA levels in serum compared to plasma, implying potential interference by platelet and white blood cells during sample preparation [83,97]. Whilst the levels of the majority of specific miRNAs are shown to be higher in serum compared to plasma, a larger number of individual miRNA can be detected from plasma compared to serum [97], highlighting the influence of sample processing procedures on miRNA detection and analysis. 

**Figure 2:**
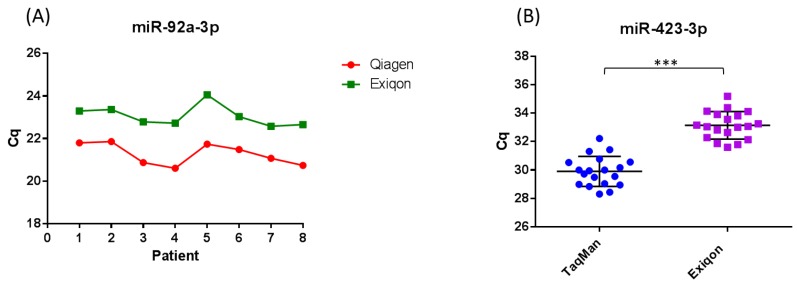
Evaluation of the miRNeasy (Qiagen) and miRCURY (Exiqon) extraction kits. (**A**) Eight healthy blood donors were used. RNA was extracted from 200 µL serum using the miRNeasy RNA isolation kit (Qiagen) and miRCURY RNA isolation kit Biofuids (Exiqon) following the manufacturer’s instructions, and eluted in 14 µL and 50 µL of RNase free water respectively. TaqMan microRNA quantitative reverse transcription polymerase chain reaction (qRT-PCR) (Applied Biosystems, Waltham, MA, USA) was carried out against miR-423-3p, a normalizer recommended by Exiqon. Cq values for miR-423-3p obtained from RNA extracted using the miRNeasy RNA isolation kit (Red) verses Cq values obtained from RNA extracted using the miRCURY RNA isolation kit Biofluids (Green) are displayed. (**B**) Eighteen healthy blood donors were used. RNA was extracted from 200 µL serum using the miRCURY RNA isolation kit Biofluids (Exiqon). TaqMan microRNA qRT-PCR and miRCURY™ LNA™ Universal RT microRNA polymerase chain reaction (PCR) (Exiqon) was carried out against miR-423-3p. Cq values for miR-423-3p obtained using the TaqMan chemistry are displayed in blue and the Cq values for miR-423-3p obtained using the SYBR-Green based (Exiqon) chemistry are displayed in yellow (see also Supplementary data). *** *p* < 0.001.

The method used to extract RNA from serum and plasma varies between studies on circulating miRNA biomarkers for melanoma, and it has been demonstrated that the extraction method used can influence the yield and distribution of miRNA detected [74,85,86,88,89,91,92,94]. It has been demonstrated by a number of studies, that the Qiagen miRNeasy miRNA extraction kits result in a higher yield of small RNA when compared to other commercial kits [85,86]. We demonstrated that the miRNeasy RNA isolation kit (Qiagen, Manchester, UK) and miRCURY RNA isolation kit (Exiqon, Vedbaek, Denmark) result in similar yields of RNA (Figure 2a). The difference in Cq observed between kits in Figure 2a is expected as RNA is eluted in 3.57× the amount of RNase free water using the miRCURY RNA isolation kit (Exiqon) compared to the miRNeasy RNA isolation kit (Qiagen), resulting in a lower concentration. Because there is a current lack of techniques to accurately quantify the concentration of RNA extracted from serum and plasma, it is often the case that studies input a fixed volume rather than fixed amount of RNA into detection platforms. This makes the miRNeasy RNA isolation method superior to the miRCURY RNA isolation method when referring to Cq. Studies using the miRCURY RNA isolation kit (Exiqon) could include a step to concentrate their RNA prior to detection in assays. Further, we demonstrated that consistency in the extraction efficiency between samples is achievable using both kits, accounting for normal human variation in miR-423-3p expression between samples (Figure 2a and Supplementary data). To control for kit-dependent variations in the expression of specific miRNA, studies could extract RNA from each sample using two different extraction kits. Indeed, a surprising proportion of studies on circulating miRNA biomarkers in melanoma use kits that are not optimized for the extraction of low abundance miRNAs from serum and plasma (Table 1 and Table 2). In addition, a large portion of the studies did not normalize to a non-human spike-in to control (such as *Caenorhabditis elegans* miR-39) for variations in the extraction step (Table 1 and Table 2).

The qRT-PCR chemistry used varies between studies (Tables S1 and S2), and can influence both the quantity and distribution of miRNA detected from serum and plasma samples. It has been demonstrated that SYBR-Green based qRT-PCR is less sensitive and specific compared to TaqMan qRT-PCR, yielding higher Cqs and broader variation within patient groups due to more stochastic qPCR amplification [74]. This can result in significant differences in miRNA expression between patient groups that are otherwise undetected when using TaqMan qRT-PCR. In support of these findings, we demonstrated that TaqMan microRNA qRT-PCR assays have higher detection sensitivity and specificity compared to SYBR-Green based microRNA qRT-PCR assays (Figure 2b and Supplementary data). Tan et al. [74] demonstrated that the TaqMan microRNA qRT-PCR system displays higher levels of accuracy, detection sensitivity and reproducibility compared to the miScript SYBR-Green qRT-PCR system, and appears to minimize extraction kit-dependent variation within the same set of samples. The use of different qRT-PCR chemistries could therefore lead to quantification errors and conflicting findings between studies. Most studies on circulating miRNA biomarkers in melanoma did not use high throughput profiling methods such as next-generation RNA sequencing (RNA-Seq) to identify potential biomarkers and normalizers prior to analysis using qRT-PCR (Tables S1 and S2). This means that they are sampling only a subset of miRNAs and not detecting other non-coding RNAs which could potentially be used as biomarkers, such as transfer RNA (tRNA) halves.

Normalization using a valid method is essential to minimize the impact of technical variations and inconsistencies in the quantity of RNA being input into profiling platforms. The global mean normalization methods are only valid for miRNA profiling studies where the levels of a large number of miRNAs (>50) are measured. This is because these methods are based on the assumption that only a minority of miRNAs change in levels between patient groups and also that the number of downregulated miRNAs is balanced by the number of upregulated miRNAs. Since deregulation of miRNA expression may occur in cancer, this may not always be a safe assumption. Where only a small number of miRNAs are assayed it is not valid to use a global normalization method [81,98]. Currently, there is no established single or group of endogenous normalizers that can be used for the quantification of circulating miRNAs [99]. Most circulating small non-coding RNAs that have been used as endogenous normalizers in circulating miRNA biomarkers studies appear to be invariant only in some instances (i.e., cancer specific and/or study specific), therefore endogenous normalizers should be identified for samples specific to each study using high throughput screenings and a stringent selection criteria (i.e., <1/2 Cq difference between patient groups) [81,100]. It is common to use the next best normalizer if none fall within your selection criteria, however it can be argued that this would introduce further variation between samples. Algorithms such as NormFinder can been used to identify the optimal normalization miRNA among a set of candidates (Table 1 and Table 2). A surprising number of studies on circulating miRNA biomarkers in melanoma did not provide logical reasoning for the normalization method used (Table 1 and Table 2). The most frequently used endogenous normalizer in studies investigating circulating miRNAs as biomarkers for melanoma was miR-16, however this is not a suitable as it has been demonstrated as a potential melanoma biomarker in a number of studies (Table 1 and Table 2) and can vary with stress [73]. Further, studies using this miRNA as a normalizer did not test samples for hemolysis, and miR-16 has been shown to be highly abundant in blood cells [96]. 

## 7. New Directions

A number of issues related to profiling platforms and inputs, serum and plasma preparation, RNA extraction, quality control, and endogenous normalization and statistical evaluation prevent reproducibility of results and have led to conflicting findings between studies. Therefore, it is essential to develop a series of standardized and streamlined reference procedures for quantification of circulating miRNAs from serum and plasma. Once there is more confidence in the experimental approach it will be possible to determine the heterogeneity of the disease between patients and also between different ethnic groups. Furthermore, circulating miRNAs may gain more acceptance as companion diagnostics for following the efficacy of cancer therapies.

A useful new method to use to identify RNA biomarkers is RNA-Seq. This technique offers an unbiased approach to identify diagnostic or prognostic microRNA biomarkers. Since RNA-Seq analyzes the whole transcriptome it would also allow the identification of other regulatory RNAs, such as small nucleolar RNAs (snoRNAs), long non-coding RNAs (lncRNAs), piwi-interacting RNAs (piwiRNAs), and transfer RNAs (tRNAs) as biomarkers, which are often at lower abundance than miRNAs [101]. RNA-Seq also has the potential of discovering novel miRNAs, and offers higher precision estimates of abundance, a greater dynamic range allowing for detection of more differentially expressed genes with higher fold change and detection of relatively low abundance transcripts, relative to other high-throughput methodologies. 

Other potentially useful ways forward include the identification of miRNAs/ncRNA biomarkers within circulating exosomes. Although miRNAs can be protected from degradation either by encapsulation in exosomes or by being bound to proteins, there has been increasing interest in exosomal miRNAs because of the possibility of manipulating miRNA cargoes for use in therapy. Methods for consistent isolation of exosomes have improved, although care still needs to be taken to avoid further technical variability between studies. The identification of specific exosomal miRNAs which change in levels during cancer progression may therefore shed light on their potential target RNAs in recipient cells which could be useful in determining potential drug targets.

## 8. Clinical Challenges

As well as technical considerations in identification of circulating miRNA biomarkers in melanoma, there are also clinical issues that need to be resolved before these potential biomarkers can be used as a clinical tool. It has been pointed out that it is unlikely for any miRNA which differs in levels in the serum/plasma of patients compared to controls can be derived from the tumor itself. This is because it would not appear possible for the number of cells in a small tumor to produce enough miRNAs in the total circulating volume of blood to increase/decrease the levels of specific miRNAs by a quantifiable amount. It has been calculated that, for a tumor of 0.5 cm in diameter, the cells within the tumor would need to produce 50,000 more miRNAs than the healthy tissue to yield a two-fold-level of increase in the blood circulation [102]. Therefore, for a prognostic biomarker, where changes in a small tumor at an early stage of the melanoma before the cancer has spread to the sentinel lymph nodes need to be detected, it would seem unlikely that enough miRNAs could be produced. However, there is evidence that melanoma cells can use miRNAs to signal to immune cells such as regulatory T-cells [103] provoking a response in miRNA secretion from these immune cells (see Section 4 above). Therefore, any changes in miRNA levels in melanoma patients need not necessarily be from the melanoma cells alone but could be derived from a number of cell types. 

Another challenge is identifying the origin of specific miRNAs detected in the circulating blood. It would obviously be desirable if specific miRNAs upregulated within melanoma cells (e.g., miR-222 [38]; see Section 3 above) are also found at increased levels in the serum/plasma of melanoma patients compared the healthy controls. However, the available evidence suggests that miRNAs are specifically secreted via a poorly understood pathway, therefore upregulation of a specific miRNA in the tumor does not necessarily mean that this miRNA will be found at increased levels in serum/plasma [104,105]. Deregulation of miRNA biogenesis can also occur in melanoma (e.g., by downregulation of AGO2 [106] therefore the miRNA secretion pathway may also be affected in cancer patients.

Another clinical issue in the when identifying of diagnostic/prognostic miRNA biomarkers for a particular cancer is the apparent lack of specificity of some miRNAs for particular cancers. For example, deregulation of circulating miR-210 has been associated with renal cell carcinoma [107], prostate cancer [108], glioma [109], and pancreatic cancer [110] in addition to melanoma. Therefore, it has been suggested that miRNAs apparently associated with many cancers act as danger signals to alert the immune system to general inflammation or inappropriate proliferation rather than being biomarkers specific to particular cancers. These results highlight the importance of using a group or signature of miRNAs as biomarkers for melanoma rather than relying on one miRNA, which may or may not be specific to melanoma. Note, however, that of the four papers listed above, only in one study [108], has normalization been carried out appropriately. Therefore, the issue of multiple associations with different cancers may not be as prevalent as it initially appears.

As can be seen from Tables S1 and S2, RNA-Seq has not yet been used as a non-biased discovery approach to identify diagnostic or prognostic biomarkers for melanoma. As well as the advantages described above, RNA-Seq can detect miRNA isomers, including those that have been modified by the addition of U’s and/or A’s at the 3′ end. For example, a study on placental-specific miRNAs showed that miR-498 cluster variants had varying degrees of adenylation at the 3′ end of certain miRNAs [111]. In addition, an investigation on plasma miRNAs from post-partum mothers showed that 32% of the 824 miRNAs detected (>1 count) [112]. Therefore, it would be interesting to use sufficient depth of RNA-Seq to detect any isomer variants in melanoma. Addition of U’s to RNAs is usually carried out by uridyl transferases (TUTases) which in some cases can stimulate their degradation by the exoribonuclease Dis3L2 [113,114]. Aberrant expression of TUTases and Dis3L2 has been shown in some cancers [115,116] suggesting a mechanistic link to these modified miRNAs. 

In summary, although the sources and destinations of miRNAs in the circulating blood are likely to be complex and present clinical challenges, there are likely to be many rewards in understanding this type of cell signaling in the human body. Provided that future work is carried out with sufficient rigor, it is likely to yield novel information that is not only relevant to cell signaling but also to biomarker research.

## 9. Concluding Remarks

Circulating miRNAs represent promising potential diagnostic, prognostic, and predictive melanoma biomarkers. In our review of the current state of knowledge in the field, we observed limited consistency between the circulating miRNA panels identified by different research groups, therefore we have only few potentially clinically useful diagnostics or prognostics circulating miRNA signature for melanoma. The most reliable of the published potential circulating diagnostic and prognostic miRNA biomarkers are those that have been identified by multiple studies showing the same direction of change between studies, have used an acceptable normalization method and have preferably been verified using an independent validation cohort. The diagnostic and prognostic biomarkers which fit these criteria are below.

The only miRNA of diagnostic value, in that they can distinguish between melanoma patients and healthy control individuals, that fits these criterial is miR-211-5p. MiR-211-5p was identified in a study which includes independent discovery and a validation studies and which used global mean normalization followed by selection of five endogenous miRNA normalizer selected using the RefFinder algorithm [62]. It was also identified as a diagnostic biomarker in another discovery cohort using median normalization [69]. It is upregulated in all stages of melanoma compared to healthy controls, therefore it would be interesting to take this miRNA forward into clinical trials.

For those miRNAs with prognostic potential, in that they can distinguish between non-metastatic and metastatic melanoma patients and/or correlate with disease progression and poor prognosis, only two studies carefully considered normalization [64,68] and in only three cases was a validation cohort used to verify their results [63,68,82]. According to our criteria above, only miR-150 can be considered as a potential prognostic biomarker. MiR-150 was upregulated in stage III/IV melanoma patients compared to stage I/II [68] and upregulation of miR-150 correlates with melanoma recurrence and poor prognosis [63,68]; therefore it would be interesting to take this miRNA forward into a clinical trial. Whilst a number of circulating miRNAs have been identified as potential prognostic biomarkers for melanoma (Table 2), it is essential to repeat these studies once a series of standardized and streamlined reference procedures for circulating miRNA quantification have been developed, to allow reproducibility of results. Only then may some of these miRNAs be taken forward into clinical trial as potential prognostic biomarkers for melanoma progression. 

## Figures and Tables

**Table 1:** Potential diagnostic circulating miRNA biomarkers for melanoma. This table summarizes the miRNAs found to be differentially expressed in the circulation of melanoma patients relative to healthy controls (Stage 0), highlighting the technical variables that may lead to the lack of consistency between studies. Pastel blue rows refer to discovery/training patient cohorts. Pastel orange rows refer to independent validation patient cohorts. MicroRNAs showing the same direction of change between multiple studies and/or have been verified using both a discovery and validation cohort are shown in green and those that additionally have used an acceptable normalization method and have been verified using an independent validation cohort are shown in green and are underlined. MicroRNAs showing a different direction of change between studies are shown in red. Exogenous spike-in controls are shown in blue. Further details are given in Table S1.

Ref	miRNA Up-Regulated	miRNA Down-Regulated	Sample Type	Sample Distribution	Normalization Method
[60]	30 miRNAs	21 miRNAs	Blood cells	Stages I/II (1), II (7), III (4), IV (8); unknown stage (4); healthy (20)	small nucleolar RNA 48 (RNU48)
	16 miRNA signature including: miR-186, let-7d, miR-18a, miR-145, miR-99a.	16 miRNA signature including: miR-17	Blood cells	Stages II (1), III (2), IV (7), V (1); healthy (20)	small nucleolar RNA 48 (RNU48)
[61]	MEL38: 19 miRNAs (including miR-301a-3p, miR-424-5p, miR-27a-3p)	MEL38: 19 miRNAs (including miR-205-5p)	Plasma	Stages I (4), II (18), III (4) and IV (4)	cel-miR-254 and osa-miR-414
[62]	13 miRNA (including miR-211-5p)	40 miRNA	Serum	Stages 0 (4), I (11), II (17), III (11), IV (9); healthy (30)	cel-miR-39, cel-miR-54, cel-miR-238; Global mean normalization and RefFinder (5 miRNAs)
	miR-193b-3p, miR-720, miR-205-5p, miR-126-5p, miR-211-5p, miR-206, miR-550a-3p, miR-627-5p, miR-629-5p	miR-204- 5p, miR-182-5p, miR-301a-3p, miR-200c-3p, miR-28-5p, miR-27a-3p, miR-197-3p, miR-374a-5p	Serum	Stages 0 (4), I (11), II (17), III (11), IV (9); healthy (30)	cel-miR-39, cel-miR-54, cel-miR-238; Global mean normalization and RefFinder (5 miRNAs)
[64]	miR-15b-5p, miR-149-3p, and miR-150-5p	miR-193a-3p and miR-524-5p	Plasma	Stage I–II (10), III (10), IV (10); healthy (32)	Global mean normalization and NormFinder
[66]	12 dysregulated	12 dysregulated	Serum	Stage IV males (7); healthy males (4)	NormFinder & geNorm (miR-320a)
	not applicable	miR-29c-5p and miR-324-3p	Serum	Stage IV males and females (28); healthy males and females (10)	NormFinder & geNorm (miR-320a)
[68]	miR-16 and miR-211	miR-4487, miR-4706, miR-4731, miR-509-3p, miR-509-5p	Serum	Stages I/II (86), III (50), IV (119); healthy (102), healthy high nevus count (12), 16 history of melanoma	cel-miR-39; median normalization
[69]	not applicable	miR-125b	Serum and exosomes	Advanced melanoma (21: 71% with metastases), 16 disease-free, 19 healthy	miR-16; cel–miR-54
[70]	miR-20a, a miR of the 17–92 complex, and miR-125b, miR-146a, miR-155, miR-181a, miR-223	not applicable	Plasma	Uveal melanoma (6), healthy donors (26), donors (26)	cel-miR-39

**Table 2:** Potential prognostic circulating miRNA biomarkers for melanoma. This table summarizes the miRNAs found to be differentially expressed in the circulation of metastatic melanoma patients compared to non-metastatic melanoma patients, and/or those whose up- or down-regulation correlates with poor prognosis, highlighting the technical variables that may lead to the lack of consistency between studies. Pastel blue rows refer to discovery/training patient cohorts. Pastel orange rows refer to independent validation patient cohorts. Stage 0 = Healthy controls. MiRNAs showing the same direction of change between multiple studies and/or have been verified using both a discovery and validation cohort are shown in green and those that additionally have used an acceptable normalization method and have been verified using an independent validation cohort are shown in green and are underlined. MiRNAs showing a different direction of change between studies are shown in red. Exogenous spike-in controls are shown in blue. Further details are given in Table S2.

Ref.	miRNA Up-Regulated	miRNA Down-Regulated	Sample Type	Sample Distribution	Normalization Method
[61]	MEL18	MEL18	Plasma	Stages I (4), II (18), III (4) and IV (4)	cel-miR-254 and osa-miR-414
[62]	13 miRNA (inc miR-193b-3p, miR-720)	40 miRNA	Serum	Serum, serum pools, melanoma and normal tissue, cell lines, whole blood	cel-miR-39, cel-miR-54, cel-miR-238; Global mean normalization and RefFinder (5 miRNAs)
	miR-193b-3p, miR-720	-	Serum	Stages 0 (4), I (11), II (17), III (11), IV (9); healthy (30)	cel-miR-39, cel-miR-54, cel-miR-238; Global mean normalization and RefFinder (5 miRNAs)
[63]	miR-199a-5p, miR-150, miR-424	miR-15b, miR-33a	Serum	Stages I (34), II (13), III (8); recurred stages I (5), II (7), III (13)	Median normalization
	miR-199a-5p, miR-150, miR-424	miR-15b, miR-33a	Serum	Stage I (10), II (16), III (4); recurred stages I (0), stage II (12), III (8)	Median normalization
[64]	-	miR-200c-3p	Plasma	Stage I–II (10), III (10), IV (10); healthy (32)	Global mean normalization and NormFinder
[67]	miR-15b, miR-425, miR-150	miR-30d	Serum	201 stages I, II and III recurred and non-recurred	miR-30c and miR-181a NormFinder
	miR-15b, miR-425, miR-150	miR-30d	Serum	82 stages I, II and III recurred and non-recurred	miR-30c and miR-181a NormFinder
[71]	-	miR-16	Serum	Stages I (30), II (30), III (30) and IV (30); cancer-free (120)	cel-miR-39
[74]	-	miR-206	Serum	Stages I/II (20), III/IV (40)	RNA U6
[75]	miR-21	-	Plasma	Stages 0–II (12), III (10), IV (4); 3-year recurrence-free-survival (4), preoperative postoperative; benign (2) & dysplastic nevus (4)	Not specified
[76]	miR-221	-	Serum	Stages I, II, III and IV (90), healthy controls (not specified)	cel-miR-54
[77]	miR-221	-	Serum	Stages I/II (27), III/IV (45); healthy (54)	miR-16, cel-miR-54
[81]	miR-210	-	Plasma	Stages III (20), IV (26); healthy (6)	Standard curves generated by using five serially diluted melanoma cell RNA
	miR-210	-	Plasma	Stages III (60), IV (70) disease-free; stage III (46 recurred <2 years/42 recurred >5 years); healthy (35)	Standard curves generated by using five serially diluted melanoma cell RNA

## Supplementary Materials

The following are available online at http://www.mdpi.com/2218-273X/8/2/21/s1, Supplemental Methods, Supplemental Table S1: Potential diagnostic circulating miRNA biomarkers for melanoma, Supplemental Table S2: Potential prognostic circulating miRNA biomarkers for melanoma, Supplemental Table S3: List of miRNAs included in miRNA biomarker signatures.
