# Supplementary material for: Circulating MicroRNA Biomarkers in Melanoma: Tools and Challenges in Personalised Medicine
Source: Biomolecules. 2018 Apr 26;8(2):21. doi: 10.3390/biom8020021 (PMC6022922; doi:10.3390/biom8020021)
Supplement: Supplementary file 1 [file biomolecules-08-00021-s001.zip › Supplemental table 1 - Diagnostic biomarkers.docx]

Supplemental Table 1: Potential diagnostic circulating miRNA biomarkers for melanoma. This table summarises the circulating miRNAs found to be differentially expressed in the circulation of melanoma patients relative to healthy controls (Stage 0), highlighting the technical variables that may lead to the lack of consistency between studies. Pastel blue rows refer to discovery/training patient cohorts. Pastel orange rows refer to independent validation patient cohorts. MiRNAs showing the same direction of change between multiple studies and/or have been verified using both a discovery and validation cohort are shown in green. MiRNAs that have been identified by multiple studies showing the same direction of change between studies, have used an acceptable normalisation method and have been verified using an independent validation cohort are shown in green and are underlined. MiRNAs showing a different direction of change between studies are shown in red. Exogenous spike-in controls are shown in blue. Abbreviations for assays are as follows: TaqMan qRT-PCR = TaqMan microRNA qRT-PCR assays (Applied Biosystems); miScript qRT-PCR = miScript SYBR Green PCR Kit (Qiagen); miRCURY qRT-PCR = miRCURY LNA Universal RT microRNA qRT-PCR assay (Exiqon); 1066 miRNA qPCR array = miScript 1066 miRNA qPCR array platform (Qiagen, miRBase v.16); 866 miRNA microarray platform = 866 miRNA microarray platform (GRTA); multiplexed nCounter microRNA Assay V3 = multiplexed nCounter microRNA Assay V3 (Nano-String). Abbreviations for RNA extractions kits are as follows: miRNeasy Kit = miRNeasy RNA Isolation kit (Qiagen); miRNeasy serum/plasma kit = miRNeasy serum/plasma kit (Qiagen); miRNeasy FFPE Kit = miRNeasy FFPE Kit (Qiagen).

| **Reference** | **miRNA**  **up-regulated** | **miRNA**  **down-regulated** | **Sample type** | **Sample distribution** | **Samples checked for Haemolysis** | **Plasma/ serum preparation protocol** | **RNA Extraction kit** | **Normalisation method** | **RT** | **qPCR** | **Other detection method** |
| --- | --- | --- | --- | --- | --- | --- | --- | --- | --- | --- | --- |
| **60** | 30 miRNAs | 21 miRNAs | Blood cells | Stages I/II (1), II (7), III (4), IV (8); unknown stage (4); healthy (20) | Not specified | not applicable | miRNeasy kit | small nucleolar RNA 48 (RNU48) | TaqMan qRT-PCR | TaqMan qRT-PCR | 866 miRNA microarray platform/ Geniom Biochip |
|  | 16 miRNA signature including: miR-186, let-7d, miR-18a, miR-145, miR-99a, miR-664, miR-501-5p, miR-378, miR-1280, miR-365, miR-1249, miR-328, miR-422a | 16 miRNA signature including: miR-17 | Blood cells | Stages II (1), III (2), IV (7), V (1); healthy (20) | Not specified | not applicable | miRNeasy kit | small nucleolar RNA 48 (RNU48) | TaqMan qRT-PCR | TaqMan qRT-PCR | Not applicable |
| **61** | MEL38**:** 19 miRNAs (including **miR-301a-3p**, **miR-424-5p**, **miR-27a-3p**) | MEL38: 19 miRNAs (including **miR-205-5p**) | Plasma | Stages I (4), II (18), III (4) and IV (4) | Not specified | Not specified | miRNeasy Serum/Plasmakit | **cel-miR-254** and **osa-miR-414** | Not applicable | Not applicable | multiplexed nCounter microRNA Assay V3 |
| **62** | 13 miRNA including **miR-211-5p** | 40 miRNA | Serum | Stages 0 (4), I (11), II (17), III (11), IV (9); healthy (30) | Yes | Incubation time 1 hr: 2000g, 15 min | miRNeasy Serum/Plasmakit | **cel-miR-39**, **cel-miR-54**, **cel-miR-238**; Global mean normalisation and RefFinder (5 miRNAs) | miScript qRT-PCR | miScript qRT-PCR /1066 miRNA qPCR array | Not applicable |
|  | miR-193b-3p, miR-720, **miR-205-5p**, miR-126-5p, **miR-211-5p**, **miR-206**, miR-550a-3p, miR-627-5p, miR-629-5p | miR-204- 5p, miR-182-5p, **miR-301a-3p**, miR-200c-3p, miR-28-5p, **miR-27a-3p**, miR-197-3p, miR-374a-5p | Serum | Stages 0 (4), I (11), II (17), III (11), IV (9); healthy (30) | Yes | Incubation time 1 hr: 2000g, 15 min | miRNeasy Serum/Plasmakit | **cel-miR-39**, **cel-miR-54**, **cel-miR-238**; Global mean normalisation and RefFinder (5 miRNAs) | miScript qRT-PCR | miScript qRT-PCR | Not applicable |
| **64** | **miR-15b-5p**, miR-149-3p, and **miR-150-5p** | miR-193a-3p and miR-524-5p | Plasma | Stage I-II (10), III (10), IV (10); healthy (32) | Not specified | 1900g, 10 min | miRNeasy Serum/Plasmakit | Global mean normalization and NormFinder | miRCURY qRT-PCR | miRCURY qRT-PCR | Not applicable |
| **Reference** | **miRNA**  **up-regulated** | **miRNA**  **down-regulated** | **Sample type** | **Sample distribution** | **Samples checked for Haemolysis** | **Plasma/ serum preparation protocol** | **RNA Extraction kit** | **Normalisation method** | **RT** | **qPCR** | **Other detection method** |
| **66** | 12 dysregulated | 12 dysregulated | Serum | Stage IV males (7); healthy males (4) | Yes | Not specified | miRNeasy kit | NormFinder & geNorm (miR-320a) | miRCURY qRT-PCR | miRCURY qRT-PCR | Not applicable |
|  | not applicable | miR-29c-5p and **miR-324-3p** | Serum | Stage IV males and females (28); healthy males and females (10) | Yes | Not specified | miRNeasy kit | NormFinder & geNorm (miR-320a) | miRCURY qRT-PCR | miRCURY qRT-PCR | Not applicable |
| **68** | **miR-16** and **miR-211** | miR-4487, miR-4706, miR-4731, miR-509-3p, miR-509-5p | Serum | Stages I/II (86), III (50), IV ( 119); healthy (102), healthy high nevus count (12), 16 history of melanoma | Not specified | 1500g, 10 min | miRNeasy FFPE kit | **cel-miR-39**; Median normalisation | TaqMan qRT-PCR | TaqMan qRT-PCR | Not applicable |
| **69** | not applicable | **miR-125b** | Serum and serum exosomes | Advanced melanoma (21: 71% with metastases), 16 disease-free, 19 healthy | Not specified | ExoQuick precipitation | miRNeasy kit | miR-16; **cel–miR-54** | TaqMan qRT-PCR | TaqMan qRT-PCR | Not applicable |
| **70** | miR-20a, a miR of the 17–92 complex, and **miR-125b**, miR-146a, miR-155, miR-181a, and miR-223 | not applicable | Plasma | Uveal melanoma (6), healthy donors (26) | Not specified | Not specified | miRNeasy kit | **cel-miR-39** | TaqMan qRT-PCR | TaqMan qRT-PCR | Not applicable |
