# Supplementary material for: Circulating MicroRNA Biomarkers in Melanoma: Tools and Challenges in Personalised Medicine
Source: Biomolecules. 2018 Apr 26;8(2):21. doi: 10.3390/biom8020021 (PMC6022922; doi:10.3390/biom8020021)
Supplement: Supplementary file 1 [file biomolecules-08-00021-s001.zip › Supplemental table 2- Prognostic biomarkers.docx]

Supplemental Table 2: Potential prognostic circulating miRNA biomarkers for melanoma. This table summarises the circulating miRNAs found to be differentially expressed in the circulation of metastatic melanoma patients compared to non-metastatic melanoma patients, and/or those whose up- or down- regulation correlates with poor prognosis, highlighting the technical variables that may lead to the lack of consistency between studies. Pastel blue rows refer to discovery/training patient cohorts. Pastel orange rows refer to independent validation patient cohorts. MiRNAs showing the same direction of change between multiple studies and/or have been verified using both a discovery and validation cohort are shown in green. MiRNAs that have been identified by multiple studies showing the same direction of change between studies, have used an acceptable normalisation method and have been verified using an independent validation cohort are shown in green and are underlined. MiRNAs showing a different direction of change between studies are shown in red. Exogenous spike-in controls are shown in blue. Abbreviations for assays are as follows: TaqMan qRT-PCR = TaqMan microRNA qRT-PCR assays (Applied Biosystems); miScript qRT-PCR = miScript SYBR Green PCR Kit (Qiagen); NCode qRT-PCR = NCode miRNA qRT-PCR kit (Applied biosystems); Mir-X qRT-PCR = Mir-X miRNA qRT-PCR SYBR Kits (Clontech); miRCURY qRT-PCR = miRCURY LNA Universal RT microRNA qRT-PCR assay (Exiqon); PerfeCTa SYBR FastMix = PerfeCTa SYBR FastMix for iQ (Quantabio); multiplexed nCounter microRNA Assay = multiplexed nCounter microRNA Assay V3 (Nano-String). Abbreviations for RNA extractions kits are as follows: miRNeasy Kit = miRNeasy RNA Isolation kit (Qiagen); miRNeasy serum/plasma kit = miRNeasy serum/plasma kit (Qiagen); miRNeasy FFPE Kit = miRNeasy FFPE Kit (Qiagen); RNeasy kit = RNeasy RNA Isolation kit (Qiagen); mirVana kit = mirVana miRNA isolation kit (LifeTechnologies); Tri-reagent = Tri-reagent (Sigma). Stage 0 = Healthy donors.

| **Reference** | **miRNA**  **up-regulated** | **miRNA**  **down-regulated** | **Sample type** | **Sample**  **distribution** | **Samples checked**  **for Haemolysis** | **Plasma/**  **serum preparation protocol** | **RNA Extraction kit** | **Normalisation method** | **RT** | **qPCR** | **Other detection method** |
| --- | --- | --- | --- | --- | --- | --- | --- | --- | --- | --- | --- |
| **61** | MEL18 | MEL18 | Plasma | Stages I (4), II (18), III (4)and IV (4) | Not specified | Not specified | miRNeasy Serum/Plasma kit | **cel-miR-254** and **osa-miR-414** | Not applicable | Not applicable | multiplexed nCounter microRNA Assay V3 |
| **62** | 13 miRNA (inc **miR-193b-3p**, **miR-720**) | 40 miRNA | Serum | Serum, serum pools, melanoma and normal tissue, cell lines, whole blood | Yes | Incubation time 1 hr: 2000g, 15 min | miRNeasy Serum/Plasma kit | cel-miR-39, cel-miR-54, cel-miR-238; Global mean normalisation and RefFinder (5 miRNAs) | miScript qRT-PCR | miScript qRT-PCR /1066 miRNA qPCR array | Not applicable |
|  | **miR-193b-3p**, **miR-720** | - | Serum | Stages 0 (4), I (11), II (17), III (11), IV (9); healthy (30) | Yes | Incubation time 1 hr: 2000g, 15 min | miRNeasy Serum/Plasma kit | cel-miR-39, cel-miR-54, cel-miR-238; Global mean normalisation and RefFinder (5 miRNAs) | miScript qRT-PCR | miScript qRT-PCR | Not applicable |
| **63** | **miR-199a-5p, miR-150, miR-424** | **miR-15b**, miR-33a | Serum | Stages I (34), II (13), III (8); recurred stages I (5), II (7), III (13) | Not specified | Centrifuged at 10°C | miRNeasy Serum/Plasma kit | Median normalization | miRCURY qRT-PCR | miRCURY qRT-PCR pick-n-mix panel | Not applicable |
|  | **miR-199a-5p, miR-150, miR-424** | **miR-15b**, miR-33a | Serum | Stage I (10), II (16), III (4); recurred stages I (0), stage II (12), III (8) | Not specified | Centrifuged at 10°C | miRNeasy Serum/Plasma kit | Median normalization | miRCURY qRT-PCR | miRCURY qRT-PCR | Not applicable |
| **Reference** | **miRNA**  **up-regulated** | **miRNA**  **down-regulated** | **Sample type** | **Sample**  **distribution** | **Samples checked**  **for Haemolysis** | **Plasma/**  **serum preparation protocol** | **RNA Extraction kit** | **Normalisation method** | **RT** | **qPCR** | **Other detection method** |
| **64** | - | miR-200c-3p | Plasma | Stage I-II (10), III (10), IV (10); healthy (32) | Not specified | 1900g, 10 min | miRNeasy Serum/Plasma kit | Global mean normalization and NormFinder | miRCURY qRT-PCR | miRCURY qRT-PCR | Not applicable |
| **67** | **miR-15b**, **miR-425, miR-150** | miR-30d | Serum | 201 stages I, II and III recurred and non-recurred | Yes | 750g, 10 min | miRNeasy kit | miR-30c and miR-181a  NormFinder | miRCURY qRT-PCR | miRCURY qRT-PCR pick-n-mix panel | Not applicable |
|  | **miR-15b**, **miR-425, miR-150** | miR-30d | Serum | 82 stages I, II and III recurred and non-recurred | Yes | 750g, 10 min | miRNeasy kit | miR-30c and miR-181a  NormFinder | miRCURY qRT-PCR | miRCURY qRT-PCR | Not applicable |
| **71** | - | **miR-16** | Serum | Stages I (30), II (30), III (30) and IV (30); cancer-free (120) | Not specified | Not specified | miRNeasy kit | **cel-miR-39** | Mir-X qRT-PCR | Mir-X qRT-PCR | Not applicable |
| **74** | - | **miR-206** | Serum | Stages I/II (20), III/IV (40) | Not specified | Not specified | miRNeasy kit | RNA U6 | NCode  qRT-PCR | NCode  qRT-PCR | Not applicable |
| **75** | miR-21 | - | Plasma | Stages 0-II (12), III (10), IV (4); 3-year recurrence free survival (4), preoperative postoperative samples; Benign (2) & dysplastic nevus (4) | Not specified | 850g, 10 minutes (x2) | Tri-reagent; RNeasy Kit; mirVana kit | Not specified | TaqMan qRT-PCR | TaqMan qRT-PCR | Not applicable |
| **76** | **miR-221** | - | Serum | Stages I, II, III and IV (90), healthy controls (not specified) | Not specified | Not specified | miRNeasy kit | **cel-miR-54** | Mir-X qRT-PCR | Mir-X qRT-PCR | Not applicable |
| **77** | **miR-221** | - | Serum | Stages I/II (27), III/IV (45); healthy (54) | Not specified | 1000g, 10 min | miRNeasy kit | miR-16, **cel-miR-54** | Mir-X qRT-PCR | Mir-X qRT-PCR | Not applicable |
| **81** | **miR-210** | - | Plasma | Stages III (20), IV (26); healthy (6) | Not specified | 1300g, 10 min; plasma processed through a 13-mm filter | Direct plasma assay | Standard curves generated by using five serially diluted melanoma cell RNA | Direct plasma assay: miRNA-specific RT primer and moloney murine leukemia virus reverse transcriptase | PerfeCTa SYBR FastMix | Not applicable |
|  | **miR-210** | - | Plasma | Stages III (60), IV (70) disease-free; stage III (46 recurred < 2 years/42 recurred > 5 years); healthy (35) | Not specified | 1300g, 10 min; plasma processed through a 13-mm filter | Direct plasma assay | Standard curves generated by using five serially diluted melanoma cell RNA | Direct plasma assay: miRNA-specific RT primer and moloney murine leukemia virus reverse transcriptase | PerfeCTa SYBR FastMix | Not applicable |
