# Supplementary material for: Circulating MicroRNA Biomarkers in Melanoma: Tools and Challenges in Personalised Medicine
Source: Biomolecules. 2018 Apr 26;8(2):21. doi: 10.3390/biom8020021 (PMC6022922; doi:10.3390/biom8020021)
Supplement: Supplementary file 1 [file biomolecules-08-00021-s001.zip › Supplemental Table 3.docx]

| Ref | name | miRNAs listed |
| --- | --- | --- |
| 60 | 30 upregulated miRNAs  21 downregulated miRNAs | miR-186, let-7d, miR-99a, miR-664, miR-18a, miR-145, miR-1280, miR-422a, miR-183, miR-1249, miR-362-3p, miR-501-5p, miR-378, miR-365, miR-151-3p, miR-342-5p, miR-328, miR-181a-2, miR-362-5p, miR-584, miR-550, miR-30a, miR-361-3p, miR-625, miR-146a, miR-30e, miR-125a-5p, miR-142-3p, miR-22, miR-199a-5p.  miR-452, miR-216a, miR-17, miR-646, miR-217, miR-621, miR-517, miR-593, let-7i, miR-330-3p, miR-767-5p, miR-20b, miR-509-3-5p, miR-519b-5p, miR-518e, miR-221, miR-214, miR-106b, miR-18a, miR-107, miR-20a. |
| 61 | MEL38: 19 miRNAs upregulated  MEL38: 19 miRNAs downregulated  MEL18: 6 miRNAs upregulated between one or more stages of melanoma progression.  MEL18: 2 miRNAs downregulated between one or more stages of melanoma progression. | miR-301a-3p, miR-424-5p, miR-27a-3p, miR-34a-5p, miR-497-5p, miR-299-3p, miR-152-3p, miR-1910-5p, miR-181b-5p, miR-548a-5p, miR-454-3p, miR-4532, miR-1537-3p, miR-1258, miR-431-5p, miR-450a-5p, miR-2682-5p, miR-337-5p and miR-154-5p which  miR-205-5p, miR-548l, miR-1269a, miR-624-3p, miR-138-5p, miR-1-5p, miR-3928-3p, miR-3131, miR-1973, miR-520d-3p, miR-548ad-3p, miR-553, miR-764, miR-1302, miR-522-3p, miR-1264, miR-1306-5p, miR-219a-2-3p, miR-4787-3p  miR-152-3p, miR-1537-3p, miR-154-5p, miR-27a-3p, miR-299-3p, miR-301a-3p.  miR-377-5p and miR-4787-3p.  The direction of change of the remaining MEL18 members was not specified. |

Supplemental Table 3: List of miRNAs included in miRNA biomarker signatures.
