# Supplementary material for: Circulating MicroRNA Biomarkers in Melanoma: Tools and Challenges in Personalised Medicine
Source: Biomolecules. 2018 Apr 26;8(2):21. doi: 10.3390/biom8020021 (PMC6022922; doi:10.3390/biom8020021)
Supplement: Supplementary file 1 [file biomolecules-08-00021-s001.zip › Supplemental Methods.docx]

Review

Circulating microRNA biomarkers in melanoma: tools and challenges in personalised medicine.

**Sophie L. Mumford ^1^, Benjamin P. Towler ^2^, Amy L. Pashler ^3^, Onur Gilleard^4^, Yella Martin ^5^, and Sarah F. Newbury *^6^.**

1 1 Medical Research Building, Brighton and Sussex Medical School, University of Sussex, Falmer, Brighton, UK. BN1 9PS ; s.mumford@bsms.ac.uk

2 Medical Research Building, Brighton and Sussex Medical School, University of Sussex, Falmer, Brighton, UK. BN1 9PS ; b.towler@bsms.ac.uk

3. Medical Research Building, Brighton and Sussex Medical School, University of Sussex, Falmer, Brighton, UK. BN1 9PS ; a.l.pashler@bsms.ac.uk

4. Pathology and Pharmacy Building at The Royal London Hospital, 80 Newark Street, London, E1 2ES ; onurgilleard@aol.com

5. Huxley Building, School of Pharmacy and Biomolecular Sciences, University of Brighton, Lewes Road. Brighton BN2 4GJ ; y.martin@brighton.ac.uk

6. Medical Research Building, Brighton and Sussex Medical School, University of Sussex, Falmer, Brighton, UK. BN1 9PS ; s.newbury@bsms.ac.uk

***** Correspondence: s.newbury@bsms.ac.uk; Tel.: +44-1273-877874

Received: date; Accepted: date; Published: date

**Supplemental data**

**Methods**

**Sample details**

Serum was obtained from 8 healthy donors at Brighton and Sussex Medical School (Falmer) and 18 patients with suspected melanoma at the Queen Victoria Hospital (East Grinstead) that tested negative for melanoma. All subjects gave their informed consent for inclusion before they participated in the study. The study was conducted in accordance with the Declaration of Helsinki. The protocol for the healthy blood donors was approved by the Brighton and Sussex Medical School Research Governance and Ethics Committee R&D Ref No.: 13/182/LLE. The protocol for the patient blood samples was approved by the Research Ethics Committee (REC) London Centre (Chelsea): REC reference: 12/LO/1527; IRAS project ID: 98638.

**Serum preparation**

6ml of blood was drawn from each study participant and collected in BD Vacutainer^®^ Plus Plastic Serum Tubes. The tubes were inverted 6 times and the blood incubated at room temperature for 30 minutes to allow coagulation followed by immediate centrifugation at 4°C for 10 min at 2000g. Serum supernatant was taken as 500μl aliquots in 1.5ml Eppendorf tubes and stored at −80°C until analysis.

**Total RNA extraction from serum samples**

Total RNA was extracted from 200μ of each of the 8 healthy donor serum samples obtained at Brighton and Sussex Medical School using both the miRNeasy RNA Isolation Kit (Qiagen) and the miRCURY RNA Isolation Kit Biofuids (Exiqon) following the manufacturer’s instructions. RNA was eluted in 14μl and 50μl respectively, of RNase free water. 2.0 x 10^8^ copies of synthetic cel-miR-39 spike-in control was added directly to the lysis buffer prior to extraction. Total RNA was extracted from 200μl of each of the 18 non-melanoma donor samples obtained at Queen Victoria Hospital using the miRCURY RNA Isolation Kit Biofuids (Exiqon) following the manufacturer’s instructions and RNA was eluted in 50μl of RNAse free water. All RNA samples were stored at −80°C until analysis.

**Quantitative Reverse Transcription PCR (qRT-PCR) analysis**

Reverse transcription against RNA extracted from the serum samples of the 8 healthy donors obtained at Brighton and Sussex Medical School and the RNA extracted from the serum of the 18 non-melanoma donors obtained at Queen Victoria Hospital was carried out using TaqMan™ MicroRNA Reverse Transcription Kit (Applied Biosystems) following the manufacturer’s instructions, scaling the reaction volume down to 5μl. RT-qPCR was carried out in a 10μl reaction containing 5μl TaqMan™ Universal PCR Master Mix (Applied Biosystems), 0.5μl TaqMan specific primer (2ng/μl) (Applied Biosystems), 3.7μl RNAse free water (Sigma) and 0.8μl cDNA. qRT-PCR against RNA extracted from the serum of the 18 non-melanoma donors obtained at Queen Victoria Hospital was also carried out using miRCURY™ LNA™ Universal RT microRNA PCR following the manufacturer’s instructions.

**Statistical analysis**

All qPCR experiments were performed in triplicate and the data are presented as Cq values. Statistical analysis (Mann–Whitney/t-test) and graphs were performed using GraphPad Prism software (GraphPad Software, San Diego, California).
